# Patrolling and Cleaning: Threat Detection and Response Behaviors of Soldiers in a Social Aphid

**DOI:** 10.3390/ani15142036

**Published:** 2025-07-10

**Authors:** Zhixiang Liu, Zhentao Cheng, Hui Zhang, Xiaolei Huang

**Affiliations:** State Key Laboratory of Agricultural and Forestry Biosecurity, College of Plant Protection, Fujian Agriculture and Forestry University, Fuzhou 350002, China; zxliu@fafu.edu.cn (Z.L.); zhentao.cheng0123@gmail.com (Z.C.); zhanghui1903@163.com (H.Z.)

**Keywords:** social aphids, soldiers, altruistic behavior, patrolling

## Abstract

Housekeeping and colony defense behaviors are crucial for social aphids, as they help maintain a habitable living environment and enhance their ecological adaptability. However, over the past decades, numerous studies have focused on housekeeping and colony defense behaviors in species living in primary hosts, but little attention has been given to the secondary host stage. This constrains a deeper understanding of the altruistic behavior of social aphids, as well as the ecological and evolutionary significance of such behavior. We employed indoor video recordings to document and analyze the behaviors displayed by the soldiers of the sugarcane wooly aphid, *C. lanigera*, on secondary hosts. *C. lanigera* soldiers continuously patrol around the colony to detect potential threats. When encountering potential threats or obstacles, soldiers actively initiate cleaning behavior. The soldiers use their frontal horns to disengage the hardened honeydew, corpses, or honeydew simulants (rock sugar) that are attached to the surface of host plant leaves. Subsequently, they transport these materials away from the colony using their frontal horns or forelegs, either discarding or flicking them directly. When soldiers identify obstacles—such as predator eggshells—as natural enemies, they attack them with their frontal horns. Our findings contribute to a broader understanding of altruistic behavior in social aphids and the evolutionary success of their sociality.

## Simple Summary

Threat response behaviors (housekeeping and colony defense) have been widely reported in social aphids inhabiting galls on primary hosts. However, little is known about whether social aphids that live exclusively on secondary hosts exhibit threat response behavior and the processes by which they perceive potential threats. This study provides a detailed description of the threat response behavior exhibited by the social aphid *Ceratovacuna lanigera* Zehntner, 1897 (Hemiptera: Aphididae: Hormaphidinae) on its secondary host. The more complete behavioral patterns include patrolling to detect potential threats (e.g., hardened honeydew, corpses, and predator eggshells), using frontal horns to remove threats, and cooperation among individuals. These findings offer deeper insights into the threat response behavior of social aphids and their ecological and evolutionary significance.

## 1. Introduction

Out of over 5100 extant aphid species, sociality has remarkably evolved in approximately 80 species, all of which are restricted to the Hormaphidinae and Eriosomatinae subfamilies [1,2]. Some social aphids exhibit host alternation, typically forming completely enclosed or aperture-bearing galls on primary hosts, which foster densely populated individuals in confined and crowded spaces, while on secondary hosts, they primarily inhabit open environments [3,4]. The threats in these two environments differ significantly. Although galls protect the aphid colonies from extreme environmental conditions and natural enemies [5], the aphid colonies also face internal threats. In the galls on primary hosts, aphids produce substantial waste, including honeydew droplets, shed skins, and corpses, which pose a serious threat to colony safety [6,7]. Unlike the waste-dominated threats in galls, aphids in open environments face compounded survival pressures, including direct exposure to abiotic extremes, natural enemies, and pathogens/parasites. The lack of gall-derived protection exacerbates these threats, severely reducing aphid survival rates [8]. In this context, the efficient detection and timely removal of potential threats are crucial for the survival and reproduction of social aphids, whether they inhabit primary or secondary hosts.

Social aphids employ multiple adaptive strategies to counter pervasive threats, including host plant utilization and the production of altruistic castes. Aphids living inside completely enclosed galls use plant tissue reabsorption to process honeydew [7,9]. Alternatively, species residing in galls with openings may produce numerous altruistic individuals, known as soldiers, which are permanently sterile or fertile in the 1st or 2nd instar. Those soldiers actively dispose of the wax-coated honeydew balls and other waste through gall openings to maintain habitat cleanliness [1,3]. For instance, *Ceratovacuna japonica* (Takahashi, 1924), *Colophina clematis* (Shinji, 1922), *Hormaphis betulae* (Mordvilko, 1901), and *Pemphigus spyrothecae* Passerini, 1860 produce a large amount of powdery hydrophobic wax, encapsulating honeydew and thereby forming wax-coated “honeydew balls”. Subsequently, the soldiers use their heads to directly expel honeydew or other waste from the gall openings [6,10,11]. While these adaptive strategies are well studied in primary hosts, threat response strategies remain poorly understood in species that have lost their primary hosts and now exclusively inhabit secondary hosts.

For example, some species from *Ceratovacuna* and *Pseudoregma* lose their primary hosts during evolution and live exclusively on secondary hosts throughout the year [12,13]. Although some species from *Ceratovacuna* have currently lost their primary host, phylogenetic studies suggest that their ancestors may have possessed a primary host and could have formed galls on *Styrax* spp. plants [14]. Evolutionarily, soldiers in the secondary host stage may retain certain physiological, morphological, and behavioral traits exhibited during the primary host stage [15]. However, to date, little is known about whether social aphids living on secondary hosts retain certain housekeeping and colony defense behaviors observed in their primary hosts to cope with potential threats.

On the other hand, the ability to perceive and rapidly eliminate potential threats is crucial for the survival of insects. Numerous studies have shown that olfaction is essential for insects, including aphids, to perceive their surrounding environment [16,17,18]. Therefore, it is highly likely that social aphids assess potential threats through the odor landscape around them. For example, soldiers of a gall-forming social aphid, *Tuberaphis styraci* (Matsumura, 1917), utilize linoleic acid to identify deceased individuals and initiate cleaning behavior [19]. The olfactory environments surrounding primary and secondary hosts of social aphids also differ significantly. In galls, the odor environment is relatively homogeneous, making it easier for altruistic individuals to sense the presence of threats and initiate clearing. However, in the open environments of secondary hosts, altruistic individuals of social aphids face a more complex odor landscape. How altruistic individuals detect potential threats and whether they exhibit similar behavioral patterns on primary and secondary hosts are worthy of further investigation.

**Figure 1:**
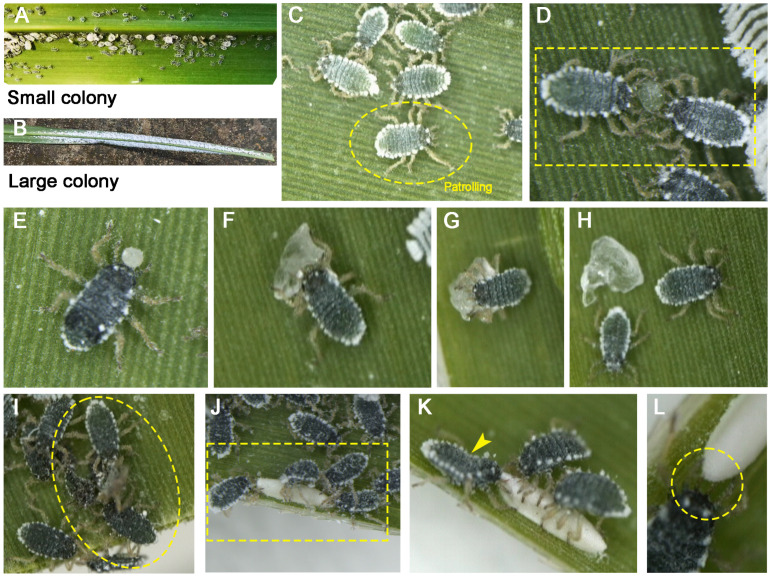
*Ceratovacuna lanigera* display altruistic behavior on secondary hosts *Miscanthus floridulus*. (**A**,**B**) The small (**A**) and large (**B**) colonies of *C. lanigera* are located on the underside of *M. floridulus* leaves. (**C**) Soldiers patrolling the surface of the leaves (from Video S1). (**D**) Two soldiers collaborating to remove hardened honeydew droplets. (**E**) The soldier grasps a droplet of honeydew on its frontal horns and moves away from the colony. (**F**–**H**) The process of a soldier transporting hardened honeydew simulants (rock sugar, from Video S3); (**F**) soldier clasping rock sugar with its fore-legs; (**G**) carrying rock sugar and moving quickly; (**H**) abandoning rock sugar. (**I**) Two soldiers cooperating to clean up corpses (from Video S4). (**J**–**L**) Soldiers attacking the eggshells of predators (from Video S5). (**J**) Four soldiers attempting to pierce the eggshell with their sharp frontal horns. (**K**) Three soldiers pricking the eggshell with frontal horns; the yellow arrows show the body bending of the soldier caused by force. (**L**) Enlarged view of soldiers attacking eggshells with their frontal horns. Task-performing soldiers were highlighted with yellow dashed markers.

Interestingly, our field observations found that soldiers of *C. lanigera* continuously walk on the underside surface of *Miscanthus floridulus* (Labill.) Warburg ex K. Schumann leaves (Figure 1A,B). Once encountering unidentified objects, they exhibited head-pushing behavior accompanied by vigorous body bending. This implies that we can use this system to investigate some previously unresolved issues. The sugarcane wooly aphid, *C. lanigera*, mainly feeds on *Saccharum* and *Miscanthus* and can generate approximately 20–25% of first-instar soldiers on secondary hosts, aiding in its defense against natural enemies [13,20]. Nevertheless, whether this species engages in housekeeping behaviors on secondary hosts remains unclear.

Taken together, what are the behavioral patterns of altruistic individuals in social aphids that respond to potential threats on secondary hosts, particularly their threat perception strategies, and do these threat-response behaviors exhibit continuity with their corresponding behaviors on primary hosts? These unresolved ecological questions highlight the need to elucidate the threat perception and response behavioral patterns of social aphids on secondary hosts. Therefore, we characterized the threat perception and response behaviors of *C. lanigera* soldiers on its secondary hosts through detailed behavioral recordings and analyses, providing partial answers to some unresolved questions. These findings not only contribute to a deeper understanding of the altruistic behavior exhibited by social aphids but also help elucidate the continuity of such behavior across both primary and secondary hosts.

## 2. Materials and Methods

### 2.1. Sample Collection

Twelve colonies of *C. lanigera* were collected along with the host plant *M. floridulus* leaves and carefully carried to the lab for detailed behavioral observations. All samples were collected from Fuzhou, Fujian Province, China, between January and March 2024. To avoid potential interference from overcrowding on video recording and data collection, all aphid colonies used in this study were collected at populations of <150 individuals. For small colonies (<150 individuals, Figure 1A), we conducted whole-colony behavior observations. Larger colonies (>400 individuals, Figure 1B) with extended aphid aggregations were divided into multiple segments to optimize behavioral monitoring in Petri dishes.

### 2.2. Behavior Observation

In the laboratory, the host plant leaves containing aphids were placed in round glass Petri dishes (9 cm in diameter) with wet filter paper for detailed behavioral analysis. Behavioral videos of patrolling and cleaning (honeydew and corpses) were recorded under the stereomicroscope (Nikon SMZ 18). Microscope magnification ranged continuously between 7.5× and 135×. To confirm the honeydew-cleaning behavior of soldiers, we crushed rock candy into ~1 mm particles, placed them near aphid colonies, and observed the soldiers’ behavioral response. All behavior videos (n = 113) and images (n = 54) were captured using the software OPLENIC (version 10.1.1). Subsequently, individuals performing different behaviors were counted (a total of 200 individuals). Given that task-performing soldiers may be interrupted by other individuals and consequently abandon their current task, we recorded both (1) individuals sustaining tasks for <60 s and (2) those persisting ≥60 s to assess behavioral task persistence in the soldiers.

### 2.3. Data Analysis

For video editing and image processing, we utilized CapCut (v7.7.0), GraphPad (v8.0.2), and Adobe Photoshop 2022, respectively. All accelerated videos (2× speed) are provided as Supplementary Materials (Video S1–S5). Detailed experimental videos can be accessed in Supplementary Materials.

## 3. Results

### 3.1. Patrolling

We observed soldiers patrolling the host leaf surfaces, their legs persistently probing while their antennae intermittently swept and tapped the leaf surfaces (Figure 1C; Video S1; n = 134). This behavior likely represents patrolling activity, during which soldiers detect potential threats or obstacles near the colony. Upon detecting such potential threats or obstacles, soldiers immediately initiate altruistic defense or housekeeping behaviors. After potential threats are removed, the same individuals resume their patrolling behavior (Video S3).

### 3.2. Cleaning Behaviors

When soldiers detect hardened honeydew near the colony, they attempt to loosen it using their frontal horns, accompanied by forceful bending of the body, and may collaborate with other individuals to remove the honeydew droplet (Figure 1D; Video S2; n = 41). After the honeydew droplet detaches from the surface of the host leaf, soldiers transport it away from the colony using their frontal horns (Figure 1E; Video S2; n = 3). To confirm the honeydew-cleaning behavior of soldiers, we positioned small artificial rock sugar particles (~1 mm in diameter) near the colonies and observed the soldiers’ behavioral responses. Upon discovering the sugar particles, soldiers initiate transporting behavior immediately (Figure 1F–H; Video S3). Initially, soldiers tightly grasp the sugar particle with their forelegs (Figure 1F), quickly move towards the edge of the colony (Figure 1G), and then discard it (Figure 1H). Furthermore, we observed soldiers flicking away the sugar crystals with their frontal horns (Video S3).

In addition to honeydew cleaning, soldiers actively perform corpse removal to maintain colony hygiene. Similarly to honeydew cleaning, soldiers use their frontal horns to remove the corpses (Figure 1I; Video S4, n = 8). Although we did not continuously monitor the behavior of cleaning corpses, drawing from our observations of soldiers engaging in honeydew cleaning, we speculate that once a corpse detaches from the surface of the host plant leaf, soldiers likely transport it to a site away from the colony or flick it aside.

### 3.3. Colony Defense

Once the soldiers identify obstacles as eggs, eggshells, and larvae of *Episyrphus* sp., they initiate colony defense. When encountering eggshells of predators, they respond by attempting to mount, pierce, and claw the eggshell (Figure 1J–L; Video S5, n = 14). Several soldiers exhibited aggressive behavior by clawing at the eggshell with their legs and forcefully piercing it with their horns (Figure 1K,L), often accompanied by body bending to extend their mid and hind legs (shown with yellow arrows in (Figure 1K). Other soldiers repeatedly mounted and attacked the eggshell (Video S5).

### 3.4. Frequency and Duration of Task Performance in Ceratovacuna Lanigera Soldiers

**Figure 2:**
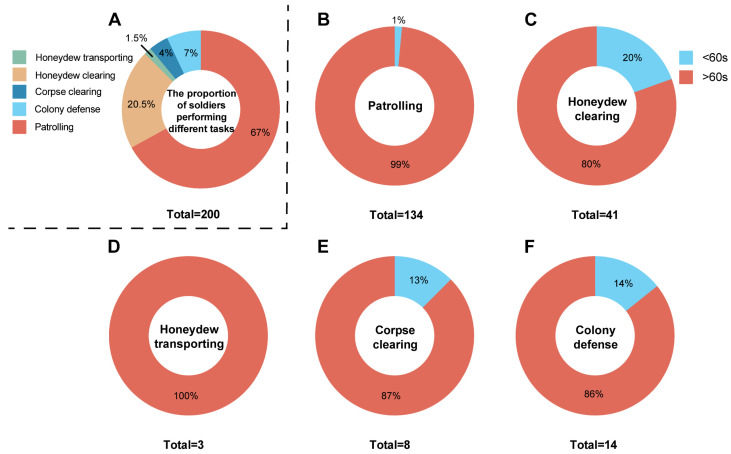
Task performance frequency and behavioral duration in *Ceratovacuna lanigera* soldiers. (**A**) Percentage distribution of soldiers performing different tasks. (**B**–**F**) Percentage distribution of different task durations in soldiers, color coding: durations < 60 s (blue), durations > 60 s (red); (**B**) Patrolling. (**C**) Honeydew clearing. (**D**) Honeydew transporting. (**E**) Corpse clearing. (**F**) Colony defense.

Preliminary behavioral sampling revealed that soldiers of *C. lanigera* specialize in five behavioral categories, displaying the following frequency distribution: 67% engaged in patrolling behaviors, 20.5% participated in honeydew clearing, while the remaining percentage performed other tasks (Figure 2A). Over 80% of soldiers continuously performed current tasks for >60 s (Figure 2B–F), with only minimal task abandonment observed. Some soldiers may engage in a single behavior for >3 min or even longer durations. For example, some individuals maintained continuous honeydew-clearing activity from initiation to successful clearing of individual honeydew particles (~3 min) (Video S2, n = 3), suggesting that soldiers may persistently engage in a specific task until the potential threat is resolved.

## 4. Discussion

Threat response has been reported in multiple social aphids; however, research has predominantly focused on the gall stage in primary hosts until now [1,3]. This study provides a detailed description of how soldiers of *C. lanigera* detect and remove threats on secondary hosts. They detect potential threats through continuous patrolling and then eliminate them using their frontal horns. It is worth noting that the patrolling behavior of *C. lanigera* soldiers may reveal an important strategy by which social aphids detect and identify threats in open environments. This provides a more comprehensive understanding of the threat responses in social aphids. However, further validation across more taxa inhabiting open environments is needed to confirm the universality of the patrolling behavior among social aphids living on secondary hosts. Another issue worthy of exploration is the origin of threat response behavior in *C. lanigera*. Although *C. lanigera* has lost its primary host, its ancestors may have formed galls on *Styrax* spp. plants [14]. We speculate that the threat response behaviors of *C. lanigera* on secondary hosts are retained from its primary host stage. This perspective is partially supported by a recent study, which suggests that the monomorphic defenders observed in the primary host stage represent a pre-existing trait among soldiers in the secondary host stage [15].

As an important aspect of threat response, honeydew cleaning is crucial for maintaining the normal survival of aphid colonies. Within galls, soldiers actively clean small balls of honeydew coated in powdery wax using their heads, legs, and posteriors, engaging in behaviors such as pushing, kicking, rolling, and backing [6]. In contrast, in open environments, *C. lanigera* soldiers remove potentially threatening honeydew particles by carrying them away from the colony or flicking them directly with their frontal horns. This may represent an adaptation of *C. lanigera* to the open environment of secondary hosts. Under field conditions, *C. lanigera* is located on the undersides of host leaves, where it uses its frontal horns to flick hardened or solidified honeydew, causing it to fall to the ground and thereby eliminating this potential threat. The frontal horns of social aphids from most species residing on secondary hosts within the genera *Ceratovacuna* and *Pseudoregma* are often regarded as significant defensive tools for colony protection [1,20]. Our study found that the frontal horn of *C. lanigera* is also used for honeydew cleaning and transport. This may reflect the principle of evolutionary conservation, whereby the frontal horn performs multiple tasks.

Cleaning up corpses or shed skins is another significant aspect of threat response, and soldiers of *C. lanigera* are responsible for this task as well. However, the detailed mechanism by which *C. lanigera* perceives corpses remains unknown. The soldiers of *Tuberaphis styraci* use linoleic acid to identify corpses and trigger cleaning behavior [15]. Similarly to the soldiers of *T. styraci*, the soldiers of *C. lanigera* may employ chemical cues to recognize corpses or other substances. Nonetheless, further research is needed to provide additional evidence supporting this hypothesis. Exploration into the role of chemical cues in *C. lanigera* soldier corpse or exuviae recognition may provide deeper insights into the altruistic cleaning behavior exhibited by *C. lanigera* soldiers.

Soldiers of social aphids also perform colony defense and display diverse defensive behavior patterns, regardless of whether soldiers reside on primary or secondary hosts [21,22,23,24,25]. *Ceratovacuna lanigera* on the host *M. floridulus* using frontal horns against the eggshells of natural enemies collectively, consistent with the findings of Aoki et al. [20]. Rapid initiation of defensive actions requires precise identification of natural enemies, but to date, it remains largely unknown whether *C. lanigera* can perceive cues related to natural enemies or other substances that need to be cleaned up, and if so, how this is achieved. Before initiating defensive behavior, soldiers physically contact substrates associated with natural enemies to assess potential threats. They may rely on sensilla, which are widely distributed across their antennae, legs, and other body parts, to detect chemical and tactile cues from the substrate surface. Therefore, chemical and tactile information may serve as key signals for soldiers to recognize natural enemies, but more evidence is needed. Future standardized experiments quantifying both soldiers’ threat-response behaviors (removal efficiency/duration) and specific enemy-recognition cues will shed light on the mechanisms triggering threat-response behavior in social aphids.

## 5. Conclusions

In conclusion, this study offers a detailed description of the threat perception-to-response behavioral sequence in soldiers of the social aphid *C. lanigera* on its secondary host. Notably, the patrolling behavior reveals the behavioral strategies by which soldiers of social aphids perceive threats in open environments. Nevertheless, our study has some limitations. First, due to experimental conditions and equipment limitations, we were unable to achieve long-term tracking and recording of aphid movements. This constraint prevented a more in-depth statistical quantification of soldiers’ efficiency in each task, allowing only preliminary quantification of the duration across different tasks. Second, the underlying mechanisms driving these behavioral patterns remain unresolved. Future studies integrating statistical analyses, omics approaches, and molecular biology techniques under standardized experimental protocols in *C. lanigera* and other social aphids exclusively inhabiting secondary hosts could provide mechanistic insights into the threat perception-response behaviors of social aphids. Such investigations will provide valuable insights into the altruistic behavior of social aphids and the evolutionary success of their sociality.

## Data Availability

The data presented in this study are available in the article. Further information is available upon request from the corresponding author.

## Supplementary Materials

The following supporting information can be downloaded at https://www.mdpi.com/article/10.3390/ani15142036/s1: Video S1: The patrolling behavior of *Ceratovacuna lanigera* soldiers; Video S2: Honeydew cleaning and transporting by *C. lanigera* soldiers; Video S3: Honeydew simulant (rock sugar) transporting by soldiers of *C. lanigera*. Video S4: Corpse removal by *C. lanigera* soldiers. Video S5: The colony defense behavior of *C. lanigera* soldiers.
